# Preservation of early Tonian macroalgal fossils from the Dolores Creek Formation, Yukon

**DOI:** 10.1038/s41598-022-10223-x

**Published:** 2022-04-13

**Authors:** Katie M. Maloney, James D. Schiffbauer, Galen P. Halverson, Shuhai Xiao, Marc Laflamme

**Affiliations:** 1 Department of Chemical and Physical Sciences, University of Toronto Mississauga, Mississauga, ON L5L 1C6 Canada; 2 Department of Geological Sciences, University of Missouri, Columbia, MO 65211 USA; 3 X-Ray Microanalysis Core, University of Missouri, Columbia, MO 65211 USA; 4 Department of Earth and Planetary Sciences/GEOTOP, McGill University, Montréal, QC H3A 0E8 Canada; 5 Department of Geosciences, Virginia Tech, Blacksburg, VA 24061 USA

**Keywords:** Geochemistry, Palaeontology

## Abstract

The rise of eukaryotic macroalgae in the late Mesoproterozoic to early Neoproterozoic was a critical development in Earth’s history that triggered dramatic changes in biogeochemical cycles and benthic habitats, ultimately resulting in ecosystems habitable to animals. However, evidence of the diversification and expansion of macroalgae is limited by a biased fossil record. Non-mineralizing organisms are rarely preserved, occurring only in exceptional environments that favor fossilization. Investigating the taphonomy of well-preserved macroalgae will aid in identifying these target environments, allowing ecological trends to be disentangled from taphonomic overprints. Here we describe the taphonomy of macroalgal fossils from the Tonian Dolores Creek Formation (ca. 950 Ma) of northwestern Canada (Yukon Territory) that preserves cm-scale macroalgae. Analytical microscopy, including scanning electron microscopy and tomographic x-ray microscopy, was used to investigate fossil preservation, which was the result of a combination of pyritization and aluminosilicification, similar to accessory mineralization observed in Paleozoic Burgess Shale-type fossils. These new Neoproterozoic fossils help to bridge a gap in the fossil record of early algae, offer a link between the fossil and molecular record, and provide new insights into evolution during the Tonian Period, when many eukaryotic lineages are predicted to have diversified.

## Introduction

Large-scale patterns in the evolution of macroalgae suggest that the Tonian Period (1000–720 Ma) hosts a significant stepwise increase in morphological diversity, which included the emergence of thallus architectures and branching morphologies^1^. This inferred diversification supports molecular phylogenetic data that predict the emergence and diversification of early eukaryotes during the late Mesoproterozoic to early Neoproterozoic^2–5^. However, the view of Proterozoic life is heavily dictated by taphonomy because the absence of widespread biomineralization limits our glimpses of Proterozoic biodiversity to exceptional taphonomic windows^6^.

The extent to which soft tissues are fossilized is sensitive to both broad-scale and fine-scale paleoenvironmental factors that can either inhibit or accelerate organismal decay^7^. The relevant factors that promote soft-tissue preservation can be divided into two categories^8,9^: (i) “facilitating factors,” such as environmental conditions that enable or favor preservation; and (ii) “driving mechanisms,” which include the mineralogical processes that constructively replicate the organism, whether by superficial mineral templating or tissue-permeating permineralization. Prominent modes of preservation during the Proterozoic include: Burgess Shale-type (BST) carbonaceous compression or kerogenization (often with accessory mineralization)^8–10^; silicification^11–15^; phosphatization^16–18^; and pyritization^8–10,19,20^. Given that taphonomic biases exert a direct control on the distribution of fossils in space and time^6^, setting realistic limits on the facilitating factors and driving mechanisms that control these taphonomic processes is essential in elucidating the natural history and paleoecology of Proterozoic life.

Several key eukaryotic fossils have been reported from late Mesoproterozoic through early Neoproterozoic strata, including the oldest identified red alga (ca. 1050 Ma *Bangiomorpha pubescens*^21^), the oldest known green alga (ca. 1000 Ma *Proterocladus antiquus*^22^), and fungal microfossils (ca. 1000 Ma *Ourasphaira giraldae*^23^)*.* While these discoveries document increasing eukaryotic diversity and complexity, biomarker studies imply that prokaryotes had remained the dominant primary producers in the oceans until at least the late Tonian Period (1000–720 Ma)^24–26^. No diagnostic eukaryotic sterane biomarkers have been reported from rocks predating ca. 800 Ma, but no biomarker data exist for the interval 1000–800 Ma^27^. Constraining the transition from cyanobacteria-dominated to eukaryotic algae-dominated ecosystems is critical because of the direct role these organisms play in the biological pump and food webs, which expanded and reorganized in response to the emergence of more complex life^2^. The discrepancies between the physical and biomarker fossil records of eukaryotes are underscored by the recent discoveries of benthic marine green macroalgal fossils in both the ca. 1000 Ma Nanfen Formation, Liaoning Province, North China^22^, and the ca. 950 Ma Dolores Creek Formation, Wernecke Mountains, Yukon, Canada, as highlighted here^28^. These recent discoveries emphasize the need to integrate biological and geological techniques to understand taphonomic biases in the biomarker and fossil records and to guide interpretations of early eukaryotic evolution and ecological expansion^5^. Specifically, identifying biases in soft-tissue fossilization is a pivotal piece of the puzzle when determining whether green algal lineages were either ecologically restricted during the Tonian, resulting in a weak biomarker signal^26,28–30^, or instead, taphonomically obscured, concealing our view of more widespread algal ecosystems. Differentiating between these two scenarios relies on recovering well-preserved fossils with enough morphological detail to identify the organisms as eukaryotic algae and sedimentological information to infer the paleoenvironmental setting. A more thorough understanding of the taphonomic pathways that promote exceptional preservation of macroalgae and other early eukaryotes will further aid in new fossil discoveries to ensure a proper representation of these clades in deep time.

**Figure 1:**
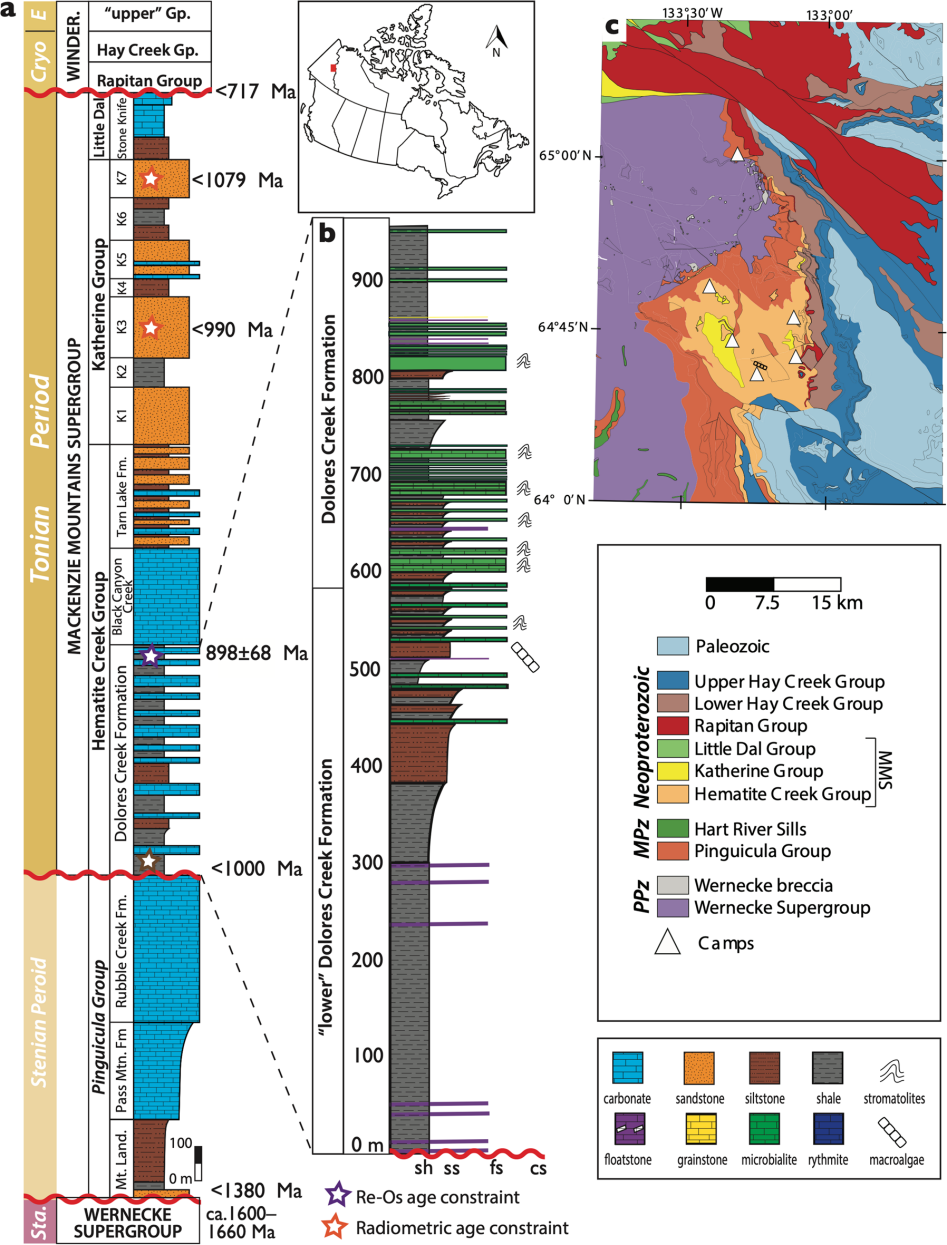
Geologic Setting. (**a**) Stratigraphy log of the Proterozoic strata that outcrop in the Wernecke Mountains with radiometric age constraints. (**b**) Enlarged interval of the fossil section of the Dolores Creek Formation including the informal “lower” Dolores Creek Formation (Yukon, Canada, 64°41′17.6′′N; 133°14′30.3′′W). (**c**) Geological map of the Tonian Mackenzie Mountains Supergroup (MMS) in the Wernecke Mountains, showing location of the fossil locality and camps where stratigraphic data was collected. Location of geologic map indicated by the red square on map of Canada. Map is modified from the Yukon Geological Survey Bedrock Geology Dataset (Yukon Geologic Survey, 2018). PPz—Paleoproterozoic; MPz—Mesoproterozoic. Gp.—Group; Fm.—Formation; Sta.—Statherian Period; Cryo—Cryogenian Period; E—Ediacaran Period, Winder.—Windermere Supergroup. K1–K7 refers to seven formations of the Katherine Group (from bottom to top): Eduni Fm., Tawu Fm., Grafe River Fm., Etagochile Fm., Shattered Range Fm., McClure Fm., and Abraham Plains Fm.

The ca. 950–775 Mackenzie Mountains Supergroup is one of the most complete Tonian successions globally and hosts well-preserved fossils that span the Neoproterozoic Era (Fig. 1)^31^. The strata outcrop in the Mackenzie Mountains (Northwest Territory, Canada) and the Wernecke Mountains (Yukon Territory, Canada) where they include the Hematite Creek, Katherine, and Little Dal groups^32^. The basal Dolores Creek Formation of the Hematite Creek Group is a mixed carbonate-siliciclastic succession, which reaches its maximum known thickness (~ 1 km) in the southern part of the basin where the macroscopic green algal fossils were discovered^28^. The fossil interval occurs above 450 m of dark grey siltstones and shales with minor debrites composed of stromatolitic floatstone. The sampled interval represents the base of a cyclic shoaling-upward sequence of fine-grained siliciclastic strata capped by stromatolite biostromes on a prograding shelf margin. The fossils are preserved on multiple bedding planes of silty shales interpreted as gravity flow deposits, indicating transport from the photic zone into relatively deeper waters^28^.

Here we report on the mode of preservation of the Dolores Creek macroalgae and associated filamentous microfossils. Our proposed taphonomic model explains the variation in the exceptional preservation of these fossils, as observed via analytical microscopic methods, and compares their mode of preservation to other similar taphonomic pathways from the late Neoproterozoic and early Paleozoic.

## Results

**Figure 2:**
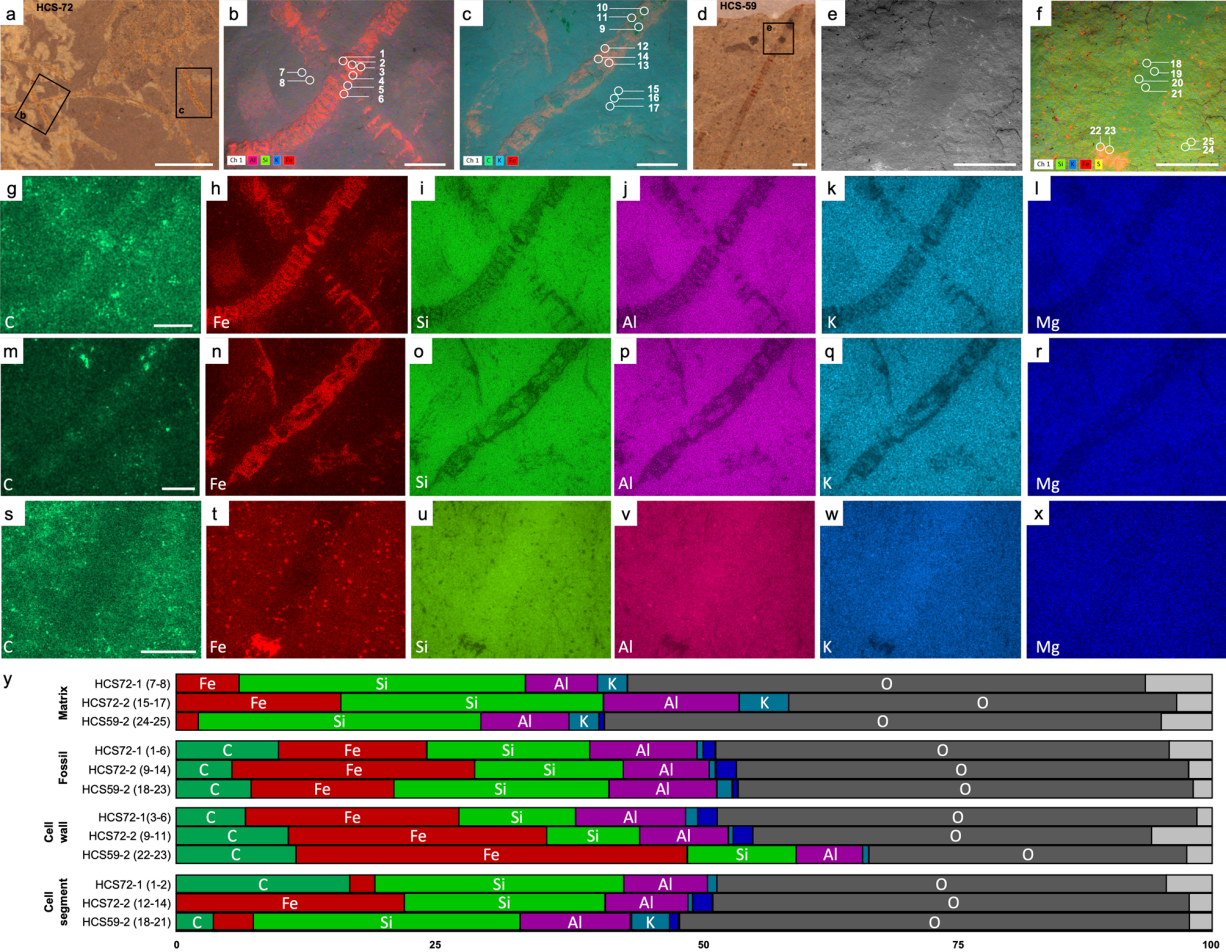
Grade 1: Well-preserved macroalgae. (**a**) Reflected light image of a well-preserved specimen (HCS-72). (**b**) SEM BSE image with EDS overlay of HCS-72-1 (**a**) (rectangle) showing the locations of sampled point data and distribution of Al, Si, K, and Fe. (**c**) SEM BSE image with EDS overlay of HCS-72-2 (**a**) (rectangle) showing the locations of sampled point data and distribution of C, K, and Fe. (**d**) Reflected light image of HCS-59. (**e**) SEM BSE image of (**d**) (rectangle). (**f**) BSE image with EDS overlay of (**d**) showing locations of sampled point data and distribution of Si, K, Fe, and S. (**g**–**l**) EDS elemental maps for specimen HCS-72-1. (**m**–**r**) EDS elemental maps for specimen HCS-72-2. (**s**–**x**) EDS elemental maps for specimen HCS-59-2. (**y**) Bar plot illustrating the concentration of each element reported as the mean relative intensity in normalized weight percent from EDS point data where Dark Blue = Mg; Light Grey = remaining elements. White scale bar = 1 mm. EM images in this figure and Figs. 3, 4 were captured using the Zeiss SmartSEM software interface, and elemental maps were collected using Bruker Esprit 2 software. All figures were assembled using Adobe Illustrator.

**Figure 3:**
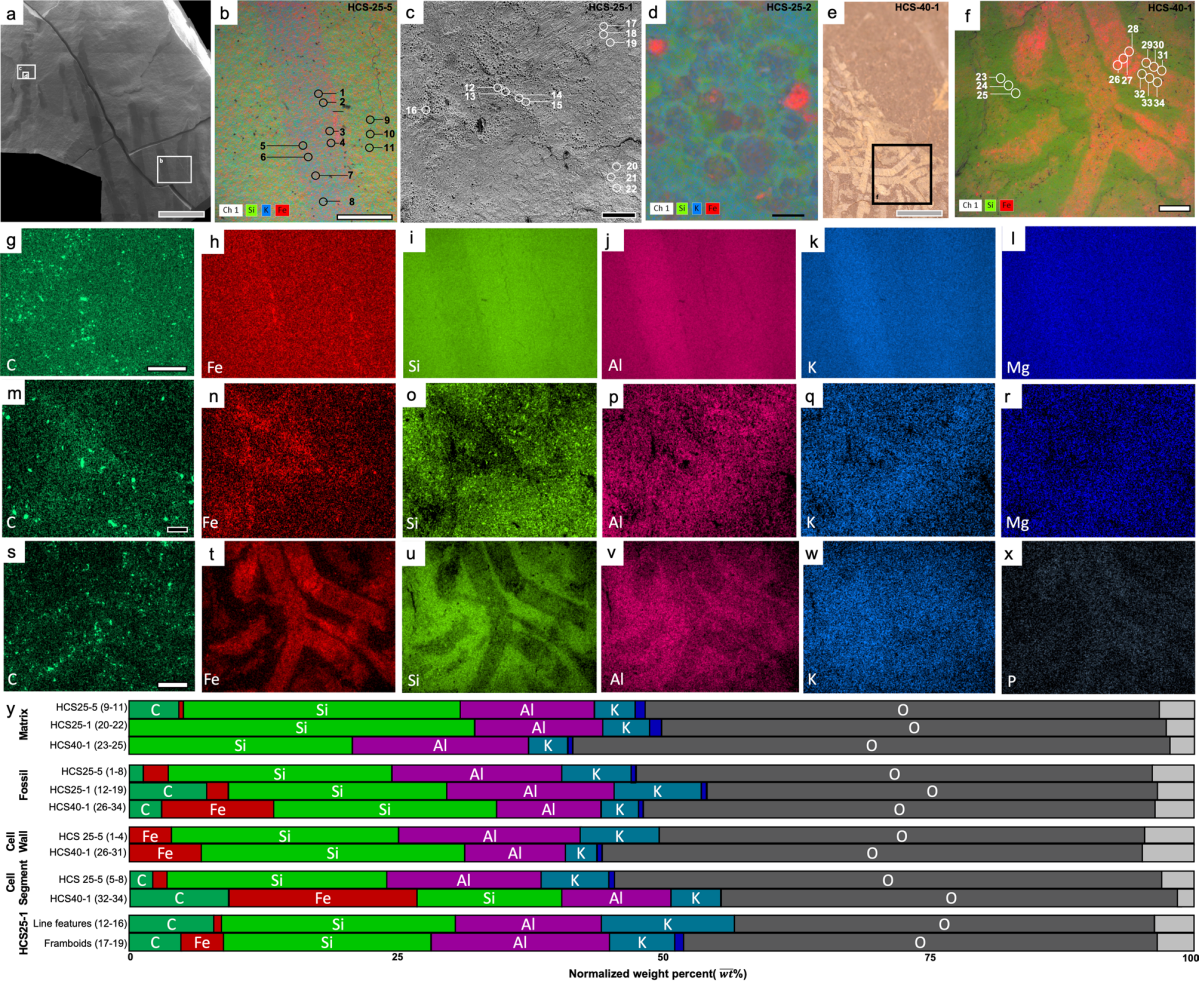
Grade 2: Moderately preserved macroalgae. (**a**) BSE image of moderately preserved specimens (HCS-25). (**b**) SEM BSE image of (**a**) (larger rectangle) with EDS overlay showing locations of sampled point data and distribution of Si, K and Fe. (**c**) BSE image with of (**a**) showing preservation textures (pits and linear features) and the locations of sampled point data. (**d**) SEM BSE image of (**a**) (smallest rectangle) with EDS overlay showing locations of sampled point data, pyrite textures, and distribution of Si, K and Fe. (**e**) Reflected light image of a moderately-preserved specimen (HCS-40). (**f**) SEM BSE image of (**e**) (rectangle) with EDS overlay showing locations of sampled point data and distribution of Si and Fe. (**g**–**l**) EDS elemental maps for specimen HCS-25-5. (**m**–**r**) EDS elemental maps for specimen HCS-25-1. (**s**–**x**) EDS elemental maps for specimen HCS-40-1. (**y**) Bar plot illustrating the concentration of each element reported as the mean relative intensity in normalized weight percent from EDS point data where Dark Blue = Mg; Light grey = remaining elements. Grey scale = 0.5 cm, white scale with black outline = 0.5 mm, black scale with white outline = 100 microns, black scale = 5 microns.

**Figure 4:**
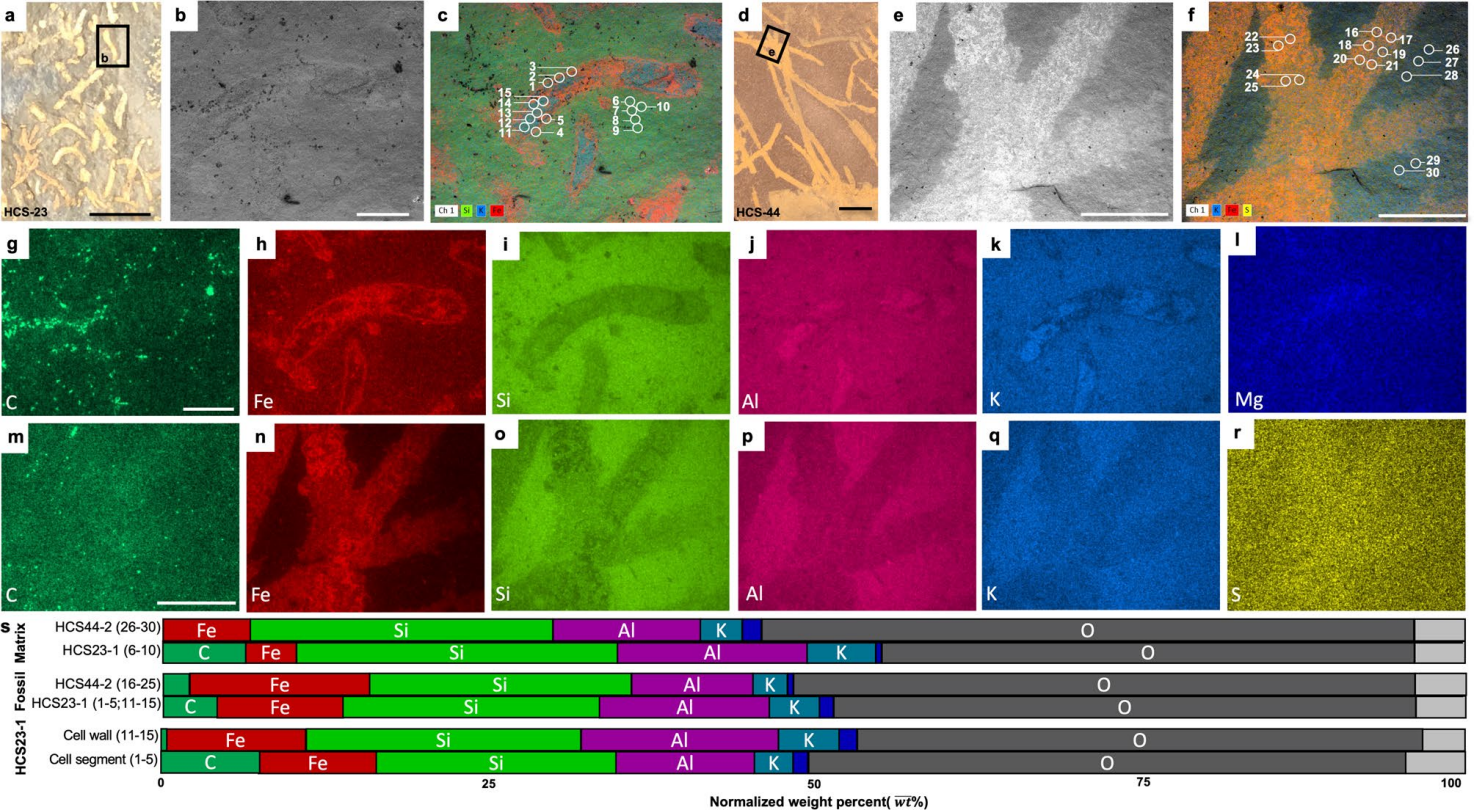
Grade 3: Poorly preserved macroalgae. (**a**) Reflected light image of HCS-23. (**b**) SEM BSE image of (**a**) (rectangle). (**c**) BSE image with EDS overlay of (**a**) showing the locations of sampled point data and distribution of Si, K, and Fe. (**d**) Reflected light image of HCS-44. (**e**) SEM BSE image of (**d**) (rectangle). (**f**) BSE image with EDS overlay of d showing locations of sampled point data and distribution of S, K and Fe. (**g**–**l**) EDS elemental maps for specimen HCS-23-1. (**m**–**r**) EDS elemental maps for specimen HCS-44-2. (**s**) Bar plot illustrating the concentration of each element reported as the mean relative intensity in normalized weight percent from EDS point data where Dark Blue = Mg; Light Grey = remaining elements. White scale = 1 mm, black scale = 5 mm.

Field sampling was conducted in 2018 and consists of 90 collected slabs and 339 observed fossil specimens. A range of preservational fidelity has been identified, which we have scored into three qualitative taphonomic grades: Grade 1, Well-preserved specimens (e.g., HCS-59, HCS-72; Fig. 2) that include a clearly defined uniseriate and filamentous cellular organization; Grade 2, moderately preserved specimens (e.g., HCS-25, HCS-40; Fig. 3) showing identifiable cellular boundaries (cross walls) and lateral cell walls (side walls) for most of the thallus; or Grade 3, poorly preserved specimens (e.g., HCS-23, HCS-44; Fig. 4), with infrequent preservation of cell walls. All elemental data herein is reported in mean normalized weight percent, $\overline{wt }$%.

### Grade 1: well-preserved specimens

**Figure 5:**
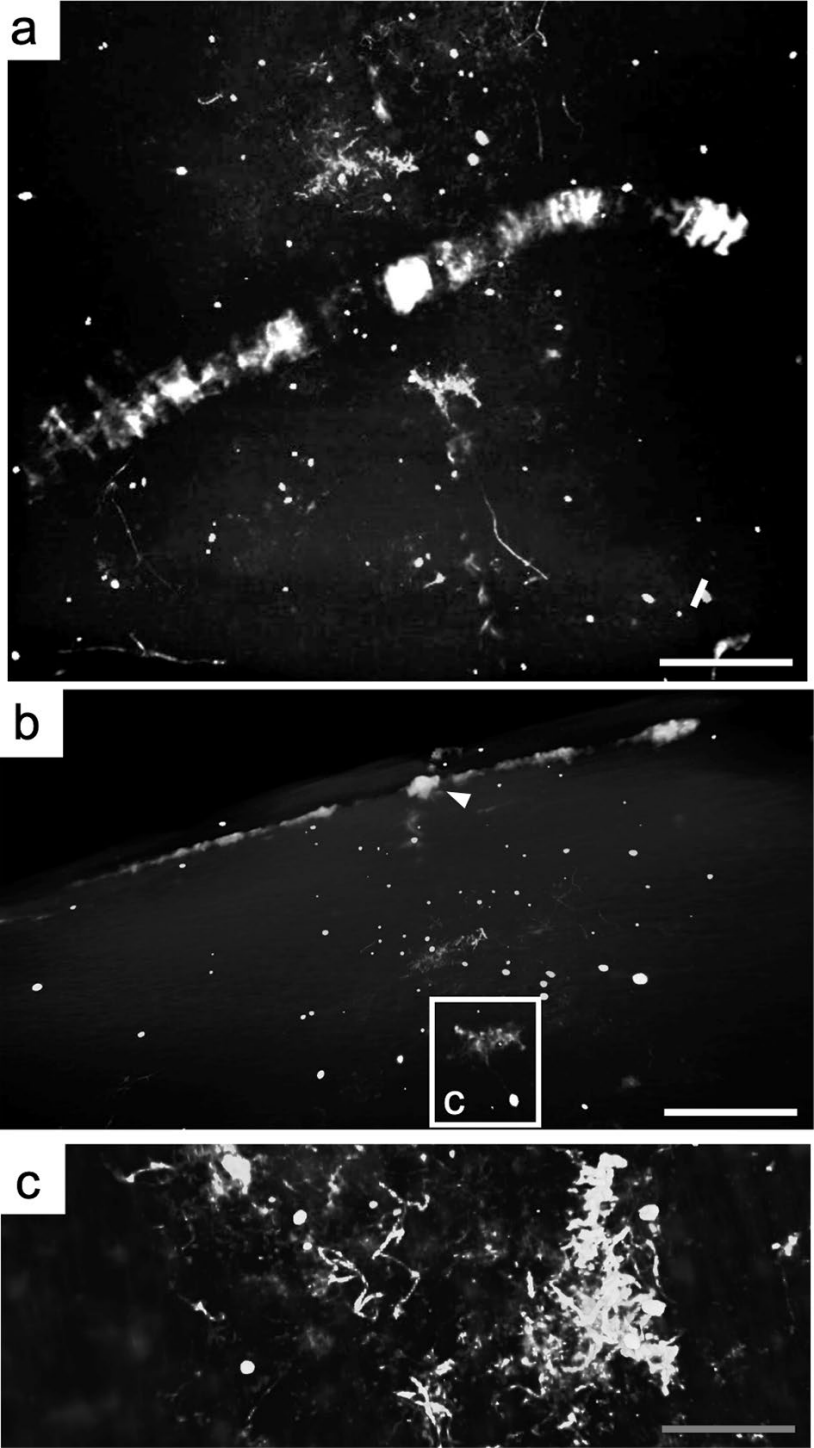
Exploratory µCT volume analysis. (**a**) µCT image of the top bedding surface of HCS-72 with high-density, ribbon-shaped material throughout the bedding planes. (**b**) µCT image of side view of HCS-72 (arrow) above unexposed, internal material. (**c**) µCT image of high-density material in (**b**) (rectangle). White scale bar = 1 mm, grey scale bar = 0.5 mm.

Compositionally, Grade 1 fossils are enriched in carbon, iron, aluminum, and magnesium relative to the host-rock matrix (Fig. 2). Cell boundaries are preserved as transverse structures defined as cross-walls between neighbouring cells while side walls are lateral structures parallel to the thallus length. The cross-walls typically appear to contain relatively more carbon than the internal cellular regions between cell boundaries. The difference in carbon concentrations between the cell walls and cell segments is subtle and varies slightly between samples (HCS72-1 cell walls = 6.43% vs. cell segment = 16.53%; HCS72-2 cell walls = 10.90% vs. cell segment = 0% [not detected]; HCS59-2 cell walls = 11.56% vs. cell segment = 3.04%; Fig. 2g,m,s,y). Conversely, iron is concentrated in the cell walls and is lowest in the cell segment (HCS72-1 cell walls = 20.61% vs. cell segment = 2.01%; HCS72-2 cell walls = 25.17% vs. cell segment = 21.93%; HCS59-2 cell walls = 37.67% vs. cell segment = 3.85%; Fig. 2h,n,t,y). Common clastic elements, silicon and potassium, are elevated in the host-rock matrix as compared to the fossils (Fig. 2y, “matrix” vs. “fossils”), while the cell segment also appears to contain slightly increased silicon compared to the cell walls (HCS72-1 cell walls = 11.31% vs. cell segment = 24.42%; HCS72-2 cell walls = 8.75% vs. cell segment = 19.03%; HCS59-2 cell walls = 10.31% vs. cell segment = 25.84%; Fig. 2i,o,u,y). Aluminum and magnesium show elevated concentrations in association with the fossils (Fig. 2j,l,p,r,v,x,y, “matrix” vs. “fossils”), with higher magnesium concentrations found in the cross-walls in HCS72-1 (cell walls = 1.92% vs. cell segment = 0.08%; Fig. 2l,y) and the cell segment of HCS59-2 (cell walls = 0.00% [not detected] vs. cell segment = 0.76%; Fig. 2r,y). Although the majority of grade 1 fossils represent two-dimensional (2D) compressions on the surface of the beds, rare instances of exceptionally well-preserved grade 1 specimens show 3D preservation (HCS-72), which was further investigated via µCT. This exploratory µCT volume analysis additionally identified thin, high-density, ribbon-shape features that are unexposed but resemble the macroalgal fossils identified herein (Fig. 5).

### Grade 2: moderately preserved specimens

Moderately preserved specimens (grade 2) were difficult to observe in backscattered SEM imaging owing to compositional similarity to the host rock matrix or dominance of thin carbonaceous material, though their cellular arrangement was still discernable (Fig. 3). Specifically, correlative imaging with light photography and ATLAS SEM mosaics was the clearest way to observe the fossil morphology (Fig. 3a). These fossils resemble carbonaceous compressions (e.g., HCS-25, HCS-40). They typically preserve a highly reflective black (or grey) surface similar to the appearance of other carbonaceous film-type fossils, such as well-known soft-tissue fossil examples from the Burgess Shale^9,33–36^. Our SEM–EDS analyses revealed limited carbon content, consistent with analyses of other thin-film carbonaceous compression fossils^35,37^.

The grade 2 specimens contain greater concentrations of aluminium (Fig. 3j,p,y) when compared to the grade 1 and 3 fossils, however, Al is not always enriched in the fossil when compared to the matrix. For example, HCS25-5 (fossil = 15.81% vs. matrix = 11.80%) and HCS25-1 fossils (fossil = 15.67% vs. matrix = 12.51%) are enriched in aluminium compared to the matrix while HCS40-1 shows the opposite trend (fossil = 9.71% vs. matrix = 16.53%, Fig. 3v,y). Grade 2 fossils show subtle enrichment of potassium in HCS25-5 (fossil = 6.71% vs. matrix = 4.69%) and HCS25-1 fossils (fossil = 8.06% vs. matrix = 3.87%, Fig. 3k,q,y) with limited carbon enrichment. The HCS-40 fossil is not enriched with potassium (Fig. 3w,y) but features a pronounced iron enrichment compared to the matrix (fossil = 10.42% vs. matrix = 0.00% [not detected], Fig. 3t,y) and other grade 2 specimens (iron in HCS25-5 fossil = 2.66%; HCS25-1 fossil = 2.54%, Fig. 3h,n,y). Pits and platy linear features (Fig. 3b–d, see Fig. 3y for “framboids” and “linear features”) were observed throughout the grade 2 fossils; platy features have increased concentrations of iron, potassium, and aluminum (Fig. 3b,j,k,p,q,y), while the pits have framboidal to cubic structures with increased iron relative to the matrix (Fig. 3d,h,n,y).

### Grade 3: poorly preserved specimens

The grade 3 specimens (e.g., HCS-23 and HCS-44) show high iron (HCS23-1, fossil = 9.88% vs. matrix = 3.77%, HCS44-2 fossil = 13.93% vs. matrix = 6.63%) similar to grade 1 fossils and lower carbon (HCS23-1 fossil = 4.07% vs. matrix = 6.48%, HCS44-2 fossil = 1.96% vs. matrix = 0.00% [not detected]) compared to the other taphonomic grades (example: grade 1 HCS72-1 fossil = 9.80%) (Fig. 4). When compared with taphonomic grade 2, there is only a slight increase in iron enrichment relative to the host-rock matrix (Fig. 4h,n,s “matrix” vs “fossil”), while taphonomic grade 1 retains higher iron. Interestingly, some of the poorly preserved specimens show localized iron along the external boundary and slight elevation within the organism (Fig. 4f,n), while others have a more noticeable increase in iron concentrated along the side wall (Fig. 4c,h). The lack of cellular detail observed in grade 3 specimens starkly contrasts with the cellular organization highlighted by iron within the filamentous thallus in grade 1 specimens. Most grade 3 specimens demonstrate uniformly distributed iron throughout the fossil, obscuring cell boundaries, although the overall ribbon-shape remains clear.

### Interpretation of taphonomic grades

The differences between the taphonomic grades should be interpreted to represent the quality of morphological characters preserved by distinct processes that may, or may not, be chemically or mineralogically related to each other. The grades therefore represent overall taphonomic fidelity—from well-preserved (grade 1), to moderately preserved (grade 2), to poorly preserved (grade 3)—but do not necessarily represent a continuum of preservation or later alteration from one grade to the next. Preservational modes, on the other hand, represent the composition of the fossil as preserved, including influences of pyritization and aluminosilicification as noted above. Specimens representing different taphonomic grades and preservational modes were recovered from the same bedding planes, with some individual fossil specimens showing variation in both grade and mode. Nevertheless, we can draw some general inferences from our observations that relate grade and mode. Grade 1 specimens preserve detailed morphology and contain the highest concentrations of carbon, and relatively higher concentrations of iron, aluminum, and magnesium when compared to the matrix. Grade 2 specimens are enriched in iron and carbon as compared to grade 1, but, of the three taphonomic grades presented, they are comparatively most similar to the matrix, perhaps owing to the common preservation via aluminosilicification. Grade 3 specimens are poorly preserved, and usually show the high iron concentrations and only a slight elevation of carbon. Although we cannot rule out the possibility that “grade 3” specimens represent a different species, this is unlikely based on the consistent morphology including a filamentous structure with uniform thallus and cell widths throughout the population regardless of grade.

## Discussion

Preservation of organic material can involve several mineralization processes^8,9^. Non-mineralized macroalgae are commonly preserved as carbonaceous compressions^1,38,39^. The outward appearance and mineralogical composition of the Dolores Creek fossils suggests that they were preserved under circumstances comparable to other Neoproterozoic macroscopic carbonaceous compressions, such as the *Chuaria-Tawuia* assemblage in the Little Dal Group^40,41^, and are otherwise generally analogous to broader BST fossil preservation^10,42–44^. The carbonaceous compressions that preserve the tubular metazoans of the Ediacaran Gaojiashan biota are also associated with pyritization and aluminosilicification^8,9,45^. These kerogenized remains are composed of recalcitrant aliphatic polymer chains resulting from the polymerization of the original organic matter^46^, but preservation can be gradational and also involve other integrated taphonomic pathways (e.g., pyritization, aluminosilicification)^8,47^. The Ediacaran Miaohe and Lantian biotas, for example, are preserved as carbonaceous compressions associated with densely packed framboidal pyrite^48^. The Dolores Creek macrofossils retain little carbonaceous material, and they are broadly preserved as aluminosilicate-templated compressions with associated iron oxides from presumed oxidative weathering of pyrite^8,36,44,49^. The observable range in taphonomic quality appears to vary with their preservational composition: the less well-preserved specimens (grade 3) tend to be preserved primarily by iron oxide coatings, whereas higher-fidelity specimens (grades 1–2) instead have consistent clay mineral coatings, with iron oxides limited to the recalcitrant cell walls (Figs. 2, 3, 4). This pattern further applies to the shiny dark grey to black clay veneers, which resemble organic carbon but are composed of aluminum, magnesium, and iron in well-preserved grade 1 specimens, as opposed to potassium and iron in moderately preserved grade 2 specimens. Textures within the fossils include micrometric linear features composed mostly of iron and aluminum platy clay mineral textures (Fig. 3d). These linear features are approximately 2 μm in width, substantially thinner than the width of longitudinal striations (~ 100 μm; see Fig. 2A,F of^28^), which were interpreted as ribbed cell wall ornamentation^28^. High densities of circular pits are interpreted to represent external molds of pyrite framboids that have been removed physically (plucked-out from slab splitting) or chemically (dissolution) (Fig. 3c,d), although chemical weathering can sometimes result in iron oxide pseudomorphs after pyrite^37,50^. Comparable filamentous fossils were observed throughout the host rock samples that have split along bedding planes, confirming the high-density features observed during µCT analysis (Fig. 5).

The preservation of the Dolores Creek macrofossils is unusual as they have relatively low carbon enrichment compared to what would typically be observed in carbonaceous compression-type fossils^49,51^. This mode of preservation involves the adsorption of ferrous iron (Fe^2+^) or aluminum by the labile tissues, which inhibits bacterial decay, allowing enough time for the original organic matter to mature to kerogens by the loss of volatile components^44,52^. Structural polysaccharides like cellulose, a common constituent of algal cell walls^53^, are likely to adsorb positively charged cations such as ferrous iron, which in turn protects them from enzymatic hydrolysis^52^ thereby interfering with their impending breakdown^54^. Ferrous iron would be readily available if the organism is buried in sediments beneath anoxic water masses, or if the resulting oxidation of organic matter by iron (III) reducing bacteria led to ferruginous pore waters. Importantly, carbon can be replaced or templated during early diagenetic mineralization, including aluminosilicification and pyritization. In the Dolores Creek macrofossils, in addition to the relative absence of carbon, there is evidence of iron enrichment on the fossil surfaces interpreted as clays and weathered pyrites, with slightly more iron on the cell walls relative to other parts of the fossil. This pattern is similar to that seen in some fossils from the Cambrian Chengjiang biota, which are also deficient in organic carbon and preserved by pyritization^37^. It is possible that the initial kerogenization of the fossils was followed by replacement by iron rich-minerals (iron oxides/hydroxides, pyrite)^34^ or that the fossils experienced extensive post-burial loss of organic carbon. Given that the fossils contain very little to no carbon, the specimens likely experienced extensive degradation and the fossils were templated by clays followed by the loss of organic material.

Clays play an important role in the protection of the labile tissues from degradation during early diagenesis^33,34,36,51,55^. It has been suggested that the clay associated with BST fossilization may have resulted from later stage metamorphism^35,56,57^; however, this is an unlikely explanation for the Dolores Creek specimens as they have only undergone, at most, up to zeolite-grade metamorphism (less than 250 °C and low pressure)^58^. On the other hand, several proposed taphonomic mechanisms invoke the role of clays in close association with the decaying carcass, whether attachment of existing clays in the environment or precipitation of clays de novo. Clays have been demonstrated to inhibit decay^33,55^ and effectively replicate labile tissues (i.e., aluminosilicification)^42,44,51^. Taphonomic experiments further demonstrate the plausibility of authigenic clay formation during (and facilitated by) organic degradation^59–61^. However, it is important to note that experimental studies have also shown that some reactive clays, like montmorillonite, can instead have a deleterious effect on preservation^47^. Based on the observed mineral composition in the Dolores Creek fossils, aluminum- or iron-rich clays were likely essential to their fossilization^55^. Additionally, smectite clays^62^ were likely involved in the preservation of grade 1 fossils, while potassium-rich illite potentially aided in preservation of the grade 2 fossils, which suggests a possible alteration product of the original Al-rich clays or heterogeneity in the paleoenvironment. Aluminum-rich kaolinite is preferentially preserved in BST fossils following metamorphic alteration, supporting the hypothesis that early diagenetic interactions with the organism protected the clay from metamorphic transformation^44^. Regardless, kaolinite can be altered to the iron-rich clay berthierine during diagenesis when Fe^2+^ is present in the pore waters^36^. The alteration of kaolinite can explain the limited aluminum enrichment yet abundant iron enrichment in the Dolores Creek macrofossils.

Pyritization also contributes to the iron enrichment observed in the fossils and specifically requires a source of organic material (i.e., the organism) buried within an environment rich with iron and sulfate^18,63^. Sulfate is converted to bisulfide (HS^–^) when sulfate-reducing bacteria oxidize organic material under normal seawater pH conditions. Details of soft tissues can be lost by the overproduction of pyrite; to ensure fossilization, pyritization must be reasonably focused on the organisms being fossilized while sulfate reduction in the surrounding matrix is supressed. Such conditions are hypothesized to be a result of limited organic availability outside of the fossil materials or otherwise low TOC levels^19,20,64^. If the process is inhibited by limited organic matter, then recalcitrant tissues can be preserved by authigenic pyrite, while cellular level details are lost during degradation and subsequent diagenesis^18,63^. To account for the exceptional preservation observed in the Dolores Creek Formation, we infer that pyritization was likely restricted early in the taphonomic process due to limited availability of sulfate^65^. This scenario contrasts with other examples (e.g., the Ediacaran Gaojiashan Lagerstätte of South China) where pyritization was unimpeded by limited sulfate or reduced iron^8,9,63^, consistent with relatively high levels of marine sulfate at this time^66^. Taphonomic grades 1 and 2 are inferred to have experienced minimal pyritization, allowing the cellular structures to be preserved in detail (except in the rare cases of 3D preservation, see below). These well-to-moderately preserved specimens were likely exposed to sulfate ions after the cellular structures were protected by aluminosilicates or stabilized as kerogen. Poorly preserved grade 3 specimens with no cellular structures potentially lacked protective clay templates^44^, based on the pervasive pyritization observed, and suffered degradation by sulfate-reducing bacteria in the presence of an adequate supply of sulfate ions. Pyritization is known to aid in the three-dimensional preservation of metazoan-grade soft tissues and refractory tissues of plants^34,63,67^, but the three-dimensional preservation observed in the Dolores Creek macrofossils illustrates that focused pyritization on cell walls can accomplish comparable taphonomic fidelity in macroalgae. A similar pattern was identified in fossil plants from the Eocene London Clay: although parenchymatous cell walls were coalified, the recalcitrant (lignified) cell walls were instead pyritized^68^. Proterozoic fossil specimens of *Grypania* and *Vendotaenia* can also be preserved by pyrite crystals in iron-rich clays and other aluminosilicate minerals^69–71^ and may share similar taphonomic pathways with the Dolores Creek fossils. The 3D preservation observed in the rare examples of Dolores Creek macroalgae appears to have been aided by the thin exterior veneering of pyrite, but the overwhelming majority of fossils are preserved as flattened specimens, indicating microenvironmental and/or taphonomic heterogeneities.

The sedimentary facies that host non-calcified macroalgae fossils in the Proterozoic are broadly similar, with deposition in shallow subtidal marine environments influenced by episodic sedimentation events^72^. Such rapid burial is essential in exceptional preservation through carbonaceous compression or related taphonomic pathways^9,73,74^. These events can transport the organisms into low oxygen settings or bury them beneath enough sediment to restrict diffusion of oxidants from overlying seawater. In addition, high sedimentation rates in settings influenced by gravity flows can dilute the sedimentary organic carbon, which is important because high contents of total organic carbon correlate with decreased preservation of Proterozoic organic walled microfossils^75^. These rapid sedimentation events are also taphonomically advantageous because they impede degradation by efficient aerobic microbes. The Dolores Creek macroalgae occur in upper slope facies, but the organisms were likely derived from the shelf margin, which was likely in the photic zone based on the abundance of stromatolites.

**Figure 6:**
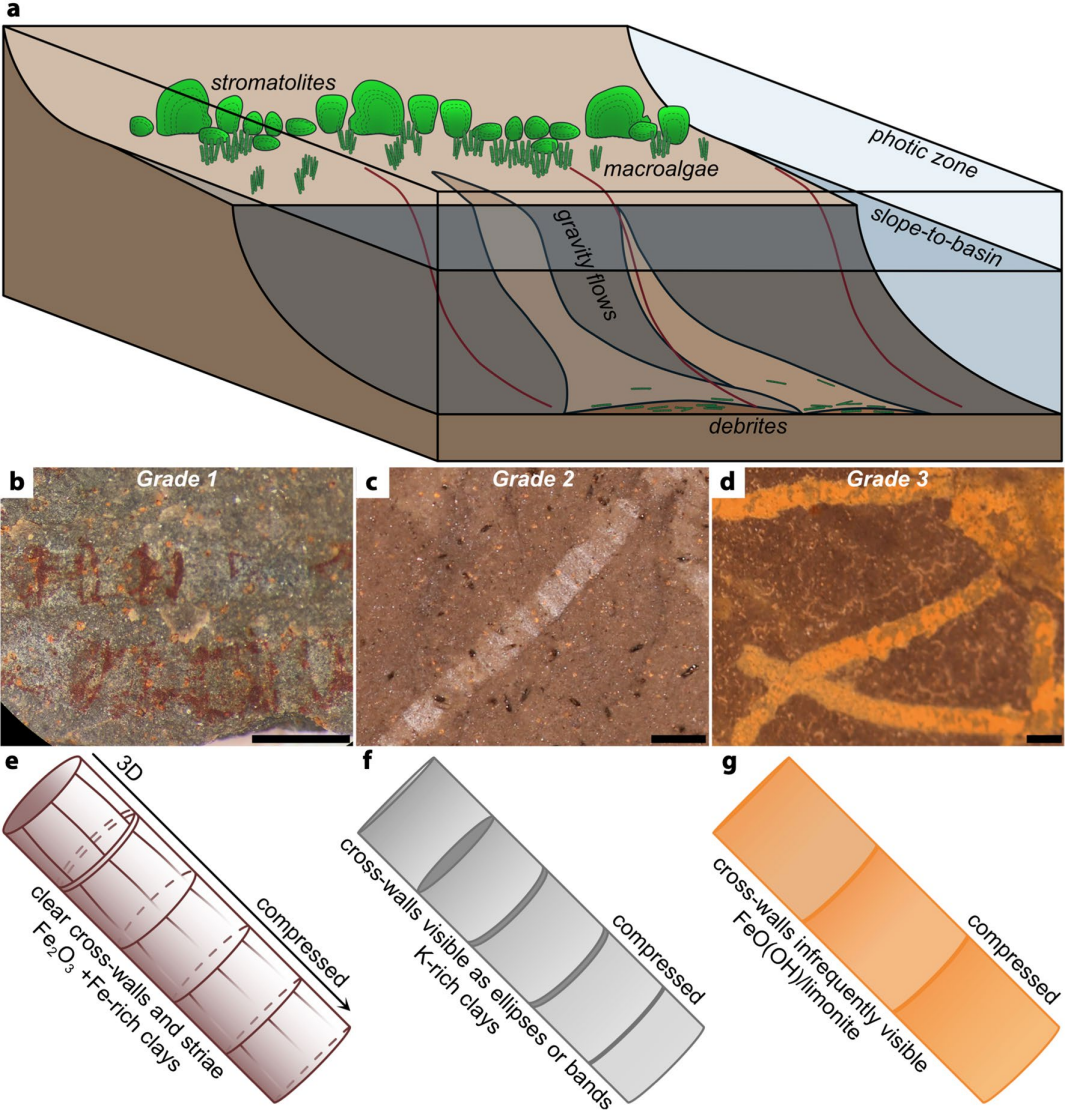
Taphonomic model for the Dolores Creek macroalgal fossils. (**a**) Depositional setting with the organisms in life position on the outer-shelf. Organisms are transported downslope and buried by gravity flows. Macroalgae decays and fossilization occurs. (**b**–**d**) Specimen photos showing variation in preservation grades 1–3. (**e**) Grade 1 fossils have pyritized cross-walls and side walls (as indicated by the presence of Fe_2_O_3_ and abundance of SO_4_^2–^ or sulfate), while the cell segments are preserved by aluminosilicification with Fe-rich clays. (**f**) Grade 2 fossils have visible cross-walls and side walls preserved by aluminosilicification with K-rich clays. (**g**) Grade 3 fossils have poorly pyritized cell boundaries because the abundant sulfate and iron in an anoxic environment resulted in the rapid precipitation of pyrite.

Following the model proposed by Schiffbauer et al.^9^, the documented range of preservation seen in the Dolores Creek fossils is hypothesized to result from a dynamic and heterogeneous environment where the availability of organic material and sulfate dictated the extent to which the specimens were pyritized (Fig. 6). We hypothesize that grade 1 specimens have clear cross walls, striae and rare 3D specimens preserved by pyrite (later oxidized to iron oxides) and iron-rich clays (Fig. 6e), although aluminum-rich clays are also implied to have been involved in early preservation before subsequent diagenetic alteration. Cross-walls are also visible in grade 2 specimens as ellipses or bands preserved by clays (likely potassium-rich clays) (Fig. 6f). Grade 3 specimens are poorly preserved by pyrite that was later oxidized to iron oxides (e.g., limonite) with cross-walls being exceedingly rare (Fig. 6g). Based on these observations, we propose the following steps to account for the exceptionally preserved fossils: (1) organisms were transported downslope by gravity flows and rapidly buried (Fig. 6a); (2) algae then experienced decay and compaction, compressing the organisms into a 2D form (with some rare 3D specimens preserved, Fig. 6e); (3) clay templating and pyritization variably preserves the fossils (Fig. 6b–g).

Biases in the fossil record can obscure the evidence of important biological events, which are crucial to the evaluation of ancient life and their paleoenvironments. The Dolores Creek macrofossils were interpreted as green algae based on their large size, putative holdfasts, and cell wall ornamentation^28^. However, many of these characteristics are absent from the moderately and poorly preserved fossils, making it impossible to differentiate between a cyanobacterial or algal origin in the poorly and moderately preserved fossils. Thus, exceptionally preserved fossils are required to accurately document the significant biological innovations among eukaryotes, including their origins^76,77^, acquisition of plastids^78^, advent of multicellularity^79^, and the onset of eukaryovory^80^. Rapid burial due to gravity flows, the availability and/or limitation of sulfate and clays, and the inhibition of decay all contribute to the exceptional preservation observed. These factors would only occur in specific depositional environments and at specific times, for example in marine, shelf margin settings with sufficient slope and sediment input to result in periodic gravity flows at a time of relatively low marine sulfate concentrations to limit pyritization. Clay formation and alteration are also influenced by the tectonic setting of the depositional environment; detrital clay deposition is favored in low energy environments, and the prevalent clay mineralogy is dictated by sediment provenance. These environmental controls are supported by our observations of the Dolores Creek Formation where macroalgal fossils were recovered from down slope debrites, whereas fossils have yet to be recovered from the stromatolitic bioherm intervals that the algae likely inhabited^28^. While this sedimentological relationship between the source of the original organisms and their site of burial and preservation highlights the likely rarity of exceptional fossils, it also provides a useful target for depositional facies that are more prone to their preservation.

Macroalgae from the Tonian Dolores Creek Formation are variably preserved by pyritization and clay templating, similar to other carbonaceous compressions from the Proterozoic, although with generally low remaining carbon content. The early templating by clays and pyrite followed rapid burial and facilitated exceptional preservation. Grade 1 organisms have the best-preserved morphology, which is critical in identifying their macroalgal affinities. Grade 2 and 3 fossils experienced greater diagenetic alteration, with grade 3 fossils having been extensively degraded by sulfate reducing bacteria in an environment where sulfate was available but not replete. Based on our results, future fossil searches should target silty shales along shelf margin to fore-slope paleoenvironments, where organic remains would be rapidly buried during episodic gravity flow events. Lagerstätten are necessary to preserve cellular level structures, which supports the hypothesis that the documented distribution of Proterozoic eukaryotic fossils is sketchy and taphonomically biased. Continued work on Tonian strata of northwestern Canada will undoubtedly contribute to further understanding of exceptional fossil preservation and the role macroalgae played in Tonian ecosystems.

## Methods

### Material and preparation

Slabs containing fossil specimens were recovered from the Dolores Creek Formation of the Mackenzie Mountains Supergroup where it outcrops near the headwaters of Hematite Creek in the Wernecke Mountains (Fig. 1). Seven in-situ beds were targeted for sampling after observing fossils in float material. Each slab contains macroalgal specimens, but the number of fossils per slab ranges from 1 to 100 s, sparsely to densely packed. Individual fossils from the in-situ beds display a range of preservation, from exceptional preservation with cellular-level details to poor preservation with only gross morphology (Figs. 2, 3, 4). All fossils analysed herein are reposited at the Royal Ontario Museum (Toronto, Ontario, Canada) and the Yukon Geological Survey (Whitehorse, Yukon, Canada). Fossils representing a range in preservation across the qualitative taphonomic grades were selected for further analyses using scanning electron microscopy (SEM), energy-dispersive x-ray spectroscopy (EDS), and tomographic X-ray microscopy (µCT).

### Analytical methods

To investigate the mineralogical differences observed in the macroalgal fossils, sample slabs exemplifying the range of taphonomic scores were selected for analysis at the University of Missouri X-ray Microanalysis Core using a Zeiss Sigma 500 variable-pressure, field emission scanning electron microscope (SEM) equipped with dual, co-planar Bruker XFlash energy dispersive X-ray spectrometers (EDS). Identical beam and chamber conditions were used for SEM imaging and EDS analyses: 20 keV beam accelerating voltage, 40 nA current, beam apertures of 60 µm (imaging) and 120 µm (EDS), a working distance of 16 mm (± 0.2 mm; flat samples allowed for minimal variation), and 20 Pa chamber pressure with a 99.999% nitrogen atmosphere. The larger aperture selection for EDS analyses serves to improve X-ray count rate, which was greater than 100 kilocounts per second in all maps and point analyses. Spatial distribution of elemental composition was determined using EDS elemental mapping (360 s live-time), supplemented with point spectral collection (60 s per point, n = 150 points over 6 slabs including both fossil and host rock points). Both spectrometers were used in tandem to help mitigate any topographic artifacts, which were likely minimal given the flat nature of the majority of the slabs and specimens. In addition, maintenance of equivalent operating conditions across all samples helps to minimize between-sample variation. EDS point data are reported in normalized weight percentages in Figs. 2, 3, 4 and in the Supplementary Materials. A high-definition 5-segment backscatter detector and a cascade current detector were used, respectively, to conduct Z-contrast backscattered (BSE) and low-vacuum secondary electron (SE) imaging. Large-area SEM mosaic images were conducted and compiled using the ATLAS workflow (Fibics, Inc.). One exceptional specimen that preserves 3D morphology (HCS-W18-72; Fig. 2) was further investigated via µCT volume imaging using a Zeiss Xradia 510 Versa. Operating conditions are as follow: 80 kV source voltage, 7 W source power, LE3 filter, 0.4 × objective, 4.5 s exposure, 2001 projections at 360 degrees, and a voxel size = 11.09 μm.

## Supplementary Information

Supplementary Video 1.

Supplementary Information 1.

## References

[1] Bykova N (2020). Seaweeds through time: Morphological and ecological analysis of Proterozoic and early Paleozoic benthic macroalgae. Precambrian Res..

[2] Sánchez-Baracaldo P, Raven JA, Pisani D, Knoll AH (2017). Early photosynthetic eukaryotes inhabited low-salinity habitats. Proc. Natl. Acad. Sci. USA..

[3] Berney C, Pawlowski J (2006). A molecular time-scale for eukaryote evolution recalibrated with the continuous microfossil record. Proc. R. Soc. B Biol. Sci..

[4] Del Cortona A (2020). Neoproterozoic origin and multiple transitions to macroscopic growth in green seaweeds. Proc. Natl. Acad. Sci. USA..

[5] Cohen PA, Kodner RB (2021). The earliest history of eukaryotic life: Uncovering an evolutionary story through the integration of biological and geological data. Trends Ecol. Evol..

[6] Muscente AD (2017). Exceptionally preserved fossil assemblages through geologic time and space. Gondwana Res..

[7] Briggs DEG (2003). The role of decay and mineralization in the preservation of soft-bodied fossils. Annu. Rev. Earth Planet. Sci..

[8] Cai Y, Schiffbauer JD, Hua H, Xiao S (2012). Preservational modes in the ediacaran gaojiashan lagerstätte: Pyritization, aluminosilicification, and carbonaceous compression. Palaeogeogr. Palaeoclimatol. Palaeoecol..

[9] Schiffbauer JD (2014). A unifying model for neoproterozoic-palaeozoic exceptional fossil preservation through pyritization and carbonaceous compression. Nat. Commun..

[10] Gaines RR, Laflamme M, Schiffbauer JD, Darroch SAF (2014). Burgess shale-type preservation and its distribution in space and time. Reading and Writing of the Fossil Record: Preservational Pathways to Exceptional Fossilization. The Paleontological Society Papers.

[11] Schopf JW (1968). Microflora of the bitter springs formation, Late Precambrian, Central Australia. J. Paleontol..

[12] Knoll AH (1985). Exceptional preservation of photosynthetic organisms in silicified carbonates and silicified peats. Philos. Trans. R. Soc. Lond. B. Biol. Sci..

[13] Xiao S, Schiffbauer JD, McFadden KA, Hunter J (2010). Petrographic and SIMS pyrite sulfur isotope analyses of Ediacaran chert nodules: Implications for microbial processes in pyrite rim formation, silicification, and exceptional fossil preservation. Earth Planet. Sci. Lett..

[14] Manning-Berg AR, Seth Wood R, Williford KH, Czaja AD, Kah LC (2019). The taphonomy of proterozoic microbial mats and implications for early diagenetic silicification. Geoscience.

[15] Slagter S, Tarhan LG, Hao W, Planavsky NJ, Konhauser KO (2020). Experimental evidence supports early silica cementation of the *Ediacara Biota*. Geology.

[16] Wilby PR, Briggs DEG (1997). Taxonomic trends in the resolution of detail preserved in fossil phosphatized soft tissues. Geobios.

[17] Xiao S, Knoll AH (1999). Fossil preservation in the Neoproterozoic Doushantuo phosphorite Lagerstatte, South China. Lethaia.

[18] Schiffbauer JD, Wallace AF, Broce J, Xiao S, Laflamme M, Schiffbauer J, Darroch SAF (2014). Exceptional fossil conservation through phosphatization. Reading and Writing of the Fossil Record: Preservational Pathways to Exceptional Fossilization. The Paleontological Society Papers.

[19] Briggs DEG, Raiswell R, Bottrell SH, Hatfield D, Bartels C (1996). Controls on the pyritization of exceptionally preserved fossils: An analysis of the Lower Devonian Hunsrück Slate of Germany. Am. J. Sci..

[20] Farrell ÚC, Laflamme M, Schiffbauer JD, Darroch SAF (2014). Pyritization of soft tissues in the fossil record: An overview. Reading and Writing of the Fossil Record: Preservational Pathways to Exceptional Fossilization. The Paleontological Society Papers.

[21] Butterfield NJ (2000). *Bangiomorpha pubescens* n. gen., n. sp.: Implications for the evolution of sex, multicellularity, and the mesoproterozoic/neoproterozoic radiation of eukaryotes. Paleobiology.

[22] Tang Q, Pang K, Yuan X, Xiao S (2020). A one-billion-year-old multicellular chlorophyte. Nat. Ecol. Evol..

[23] Loron CC (2019). Early fungi from the Proterozoic era in Arctic Canada. Nature.

[24] Isson TT (2018). Tracking the rise of eukaryotes to ecological dominance with zinc isotopes. Geobiology.

[25] van Maldegem LM (2019). Bisnorgammacerane traces predatory pressure and the persistent rise of algal ecosystems after Snowball Earth. Nat. Commun..

[26] Zumberge JA (2020). Free and kerogen-bound biomarkers from late Tonian sedimentary rocks record abundant eukaryotes in mid-Neoproterozoic marine communities. Geobiology.

[27] Brocks, J. J. & Nettersheim, B. J. Lost world of complex life: Molecular traces of our deep eukaryotic ancestors. in *Goldschmidt Meeting Abstracts* (2020).

[28] Maloney KM (2021). New multicellular marine macroalgae from the early Tonian of northwestern Canada. Geology.

[29] Brocks JJ (2017). The rise of algae in Cryogenian oceans and the emergence of animals. Nature.

[30] Nguyen K (2019). Absence of biomarker evidence for early eukaryotic life from the Mesoproterozoic Roper Group: Searching across a marine redox gradient in mid-Proterozoic habitability. Geobiology.

[31] Macdonald FA (2012). Early Neoproterozoic Basin Formation in Yukon, Canada: Implications for the make-up and break-up of Rodinia Francis. Geosci. Can..

[32] Turner EC (2011). Stratigraphy of the mackenzie mountains supergroup in the Wernecke mountains, Yukon. Yukon Explor. Geol..

[33] Butterfield NJ (1990). Organic preservation of non-mineralizing organisms and the taphonomy of the Burgess Shale. Paleobiology.

[34] Butterfield NJ (2002). *Leanchoilia* guts and the interpretation of three-dimensional structures in Burgess Shale-type fossils. Paleobiology.

[35] Gaines RR, Briggs DEG, Zhao Y (2008). Cambrian Burgess Shale-type deposits share a common mode of fossilization. Geology.

[36] Anderson RP, Tosca NJ, Gaines RR, Koch NM, Briggs DEG (2018). A mineralogical signature for Burgess Shale-type fossilization. Geology.

[37] Gabbott SE, Hou XG, Norry MJ, Siveter DJ (2004). Preservation of early Cambrian animals of the Chengjiang biota. Geology.

[38] Xiao S, Dong L, Xiao S, Kaufman AJ (2006). On the morphological and ecological history of proterozoic macroalgae. Neoproterozoic Geobiology and Paleobiology.

[39] LoDuca ST, Tetreault DK (2017). Ontogeny and reproductive functional morphology of the macroalga *Wiartonella nodifera* n. gen. n. sp. (Dasycladales, Chlorophyta) from the Silurian Eramosa Lagerstätte of Ontario, Canada. J. Paleontol..

[40] Hofmann HJ, Aitken JD (1979). Precambrian biota from the little dal group, mackenzie mountains, Northwestern Canada. Can. J. Earth Sci..

[41] Hofmann HJ, Rainbird RH (1994). Carbonaceous megafossils from the neoproterozic shaler supergroup of arctic Canada. Palaeontology.

[42] Orr PJ, Briggs DEG, Kearns SL (1998). Cambrian Burgess shale animals replicated in clay minerals. Science.

[43] Butterfield NJ (2003). Exceptional fossil preservation and the Cambrian explosion. Integr. Comp. Biol..

[44] Anderson RP, Tosca NJ, Saupe EE, Wade J, Briggs DEG (2021). Early formation and taphonomic significance of kaolinite associated with Burgess Shale fossils. Geology.

[45] Cai YP, Hua H (2007). Pyritization in the Gaojiashan Biota. Chin. Sci. Bull..

[46] Gupta NS, Briggs DEG, Pancost RD (2006). Molecular taphonomy of graptolites. J. Geol. Soc. Lond..

[47] Wilson LA, Butterfield NJ (2014). Sediment effects on the preservation of Burgess Shale-type compression fossils. Palaios.

[48] Wang W (2014). Exceptional preservation of macrofossils from the Ediacaran Lantian and Miaohe Biotas, South China. Palaios.

[49] Anderson EP, Schiffbauer JD, Xiao S (2011). Taphonomic study of Ediacaran organic-walled fossils confirms the importance of clay minerals and pyrite in Burgess Shale-type preservation. Geology.

[50] Yuan X, Xiao S, Li J, Yin L, Cao R (2001). Pyritized chuarids with excystment structures from the late Neoproterozoic Lantian formation in Anhui, South China. Precambrian Res..

[51] Anderson RP (2020). Aluminosilicate haloes preserve complex life approximately 800 million years ago. Interface Focus.

[52] Petrovich R (2001). Mechanisms of fossilization of the soft-bodied and lightly armored faunas of the Burgess Shale and of some other classical localities. Am. J. Sci..

[53] Graham LE, Wilcox LW (2000). Algae.

[54] Tejirian A, Xu F (2010). Inhibition of cellulase-catalyzed lignocellulosic hydrolysis by iron and oxidative metal ions and complexes. Appl. Environ. Microbiol..

[55] McMahon S, Anderson RP, Saupe EE, Briggs DEG (2016). Experimental evidence that clay inhibits bacterial decomposers: Implications for preservation of organic fossils. Geology.

[56] Page A, Gabbott SE, Wilby PR, Zalasiewicz JA (2008). Ubiquitous Burgess Shale-style ‘clay templates’ in low-grade metamorphic mudrocks. Geology.

[57] Becker-Kerber B (2021). Clay templates in Ediacaran vendotaeniaceans: Implications for the taphonomy of carbonaceous fossils. GSA Bull..

[58] Gibson TM (2019). Tectono-stratigraphy and facies architecture of the Tonian Hematite Creek and Katherine groups Wernecke Mountains, Yukon. Geol. Assoc. Can..

[59] Darroch SAF, Laflamme M, Schiffbauer JD, Briggs EG (2012). Experimental formation of a microbial death mask. Palaios.

[60] Newman SA (2019). Experimental preservation of muscle tissue in quartz sand and kaolinite. Palaios.

[61] Naimark E (2016). Decaying in different clays: Implications for soft-tissue preservation. Palaeontology.

[62] Odom IE (1984). Smectite clay minerals: Properties and uses. Philos. Trans. R. Soc. Lond. A.

[63] Schiffbauer JD (2020). Discovery of bilaterian-type through-guts in cloudinomorphs from the terminal Ediacaran Period. Nat. Commun..

[64] Farrell ÚC, Briggs DEG, Hammarlund EU, Sperling EA, Gaines RR (2013). Paleoredox and pyritization of soft-bodied fossils in the ordovician frankfort shale of New York. Am. J. Sci..

[65] Turner EC, Bekker A (2016). Thick sulfate evaporite accumulations marking a mid-Neoproterozoic oxygenation event Ten Stone Formation, Northwest Territories, Canada). Bull. Geol. Soc. Am..

[66] Halverson GP, Hurtgen MT (2007). Ediacaran growth of the marine sulfate reservoir. Earth Planet. Sci. Lett..

[67] Gibson BM, Schiffbauer JD, Darroch SAF (2018). Ediacaran-style decay experiments using mollusks and sea anemones. Palaios.

[68] Grimes ST (2002). Fossil plants from the Eocene London Clay: The use of pyrite textures to determine the mechanism of pyritization. J. Geol. Soc. Lond..

[69] Cohen PA (2009). Tubular compression fossils from the Ediacaran Nama Group, Namibia. J. Paleontol..

[70] Han T, Runnegar B (1992). Megascopic eukaryotic algae from the 21-billion-year-old Negaunee iron-formation, Michigan. Science.

[71] Sharma M, Shukla Y (2009). Taxonomy and affinity of Early Mesoproterozoic megascopic helically coiled and related fossils from the Rohtas Formation, the Vindhyan Supergroup, India. Precambrian Res..

[72] LoDuca ST, Bykova N, Wu M, Xiao S, Zhao Y (2017). Seaweed morphology and ecology during the great animal diversification events of the early Paleozoic: A tale of two floras. Geobiology.

[73] Cai Y, Hua H, Xiao S, Schiffbauer JD, Li P (2010). Biostratinomy of the late Ediacaran pyritized Gaojiashan Lagersttte from Southern Shaanxi, South China: Importance of event deposits. Palaios.

[74] Seilacher A, Reif W-E, Westphal F (1985). Sedimentological, ecological and temporal patterns of fossil Lägerstatten. Philos. Trans. R. Soc. Lond. B.

[75] Woltz CR (2021). Total organic carbon and the preservation of organic-walled microfossils in Precambrian Shale. Geology.

[76] Knoll AH (1992). The early evolution of eukaryotes: A geological perspective. Science.

[77] Parfrey LW, Lahr DJG, Knoll AH, Katz LA (2011). Estimating the timing of early eukaryotic diversification with multigene molecular clocks. Proc. Natl. Acad. Sci. USA..

[78] Butterfield NJ (2015). Proterozoic photosynthesis - a critical review. Palaeontology.

[79] Knoll AH (2011). The multiple origins of complex multicellularity. Annu. Rev. Earth Planet. Sci..

[80] Loron CC, Rainbird RH, Turner EC, Greenman JW, Javaux EJ (2018). Implications of selective predation on the macroevolution of eukaryotes: evidence from Arctic Canada. Emerg. Top. Life Sci..

